# The role of noncoding RNAs in cancer lipid metabolism

**DOI:** 10.3389/fonc.2022.1026257

**Published:** 2022-11-14

**Authors:** Ye Wang, Qian Li, Song Wang, Bi-jun Wang, Yan Jin, Hao Hu, Qing-sheng Fu, Jia-wei Wang, Qing Wu, Long Qian, Ting-ting Cao, Ya-bin Xia, Xiao-xu Huang, Li Xu

**Affiliations:** ^1^ Department of Gastrointestinal Surgery, The First Affiliated Hospital of Wannan Medical College, Yijishan Hospital of Wannan Medical College, Wuhu, Anhui, China; ^2^ Key Laboratory of Non-coding RNA Transformation Research of Anhui Higher Education Institution, Wuhu, Anhui, China; ^3^ Non-coding RNA Research Center of Wannan Medical College, Yijishan Hospital, Wuhu, Anhui, China; ^4^ Department of Clinical Medicine, Clinical College of Anhui Medical University, Hefei, Anhui, China

**Keywords:** noncoding RNA, lipid metabolism, tumor, diagnosis, treatment

## Abstract

Research on noncoding ribonucleic acids (ncRNAs) is mostly and broadly focused on microRNAs (miRNAs), cyclic RNAs (circRNAs), and long ncRNAs (lncRNAs), which have been confirmed to play important roles in tumor cell proliferation, invasion, and migration. Specifically, recent studies have shown that ncRNAs contribute to tumorigenesis and tumor development by mediating changes in enzymes related to lipid metabolism. The purpose of this review is to discuss the characterized ncRNAs involved in the lipid metabolism of tumors to highlight ncRNA-mediated lipid metabolism-related enzyme expression in malignant tumors and its importance to tumor development. In this review, we describe the types of ncRNA and the mechanism of tumor lipid metabolism and analyze the important role of ncRNA in tumor lipid metabolism and its future prospects from the perspectives of ncRNA biological function and lipid metabolic enzyme classification. However, several critical issues still need to be resolved. Because ncRNAs can affect tumor processes by regulating lipid metabolism enzymes, in the future, we can study the unique role of ncRNAs from four aspects: disease prevention, detection, diagnosis, and treatment. Therefore, in the future, the development of ncRNA-targeted therapy will become a hot direction and shoulder a major task in the medical field.

## Introduction

Lipids are hydrophobic molecules and include sterols, monoglycerides (MGs), diglycerides (DGs), triglycerides (TGs), phospholipids, and glycolipids. Lipids participate in the formation of membrane lipid bilayers and membrane-related protein structures. Phospholipids, glycolipids, and cholesterol are the main components of biofilms and significantly affect the fluidity of biofilms. Cholesterol is also a substrate for the synthesis of fat-soluble vitamins and steroid hormones (1). Lipids constitute the basic structure of cell membranes, and lipid anabolism is highly upregulated in human malignancies to meet the increased demand for membrane biosynthesis (2–4). Mammalian cells acquire lipids through two mechanisms, *de novo* synthesis and uptake from the environment. Acetyl-coA, as an important substrate for lipid synthesis and cholesterol synthesis, mainly comes from three sources: (1) glucose forms acetyl-CoA catalyzed by ATP citrate lyase (ACLY) through glycolysis and the Krebs cycle in mitochondria, (2) acetate is catalyzed in the cytoplasm by acetyl-CoA synthetase (ACSS) to produce acetyl-CoA, and (3) glutamate is produced by a series of reactions in the cytoplasm or mitochondria to citrate, which is catalyzed by ACLY to acetyl-CoA. Lipid uptake, storage, and fat formation are evident in many cancer types and contribute to rapid tumor growth. Lipid metabolism reprogramming is a newly discovered marker of malignant tumors (5–9).

With the continuous development of new research fields, researchers have realized that lipid metabolism is not just a self-regulation network. Technical developments in the human genome have revealed that noncoding RNAs are a new class of regulators in lipid metabolism (10, 11). Noncoding RNA is involved in tumor lipid metabolism through three ways: (1) as endogenous competing RNAs (ceRNAs) to regulate downstream mRNA stability, (2) miRNAs directly target mRNA of lipid-metabolizing enzymes, and (3) noncoding RNAs act as molecular decoys for RNA-binding proteins (RBPs). In this review, we have highlighted the regulating effects and mechanisms of noncoding RNAs on lipid metabolism and signaling, which may help advance the comprehension of the detailed modulatory networks of lipid metabolism and afford a better theoretical foundation for clinical diagnosis and treatment of metabolic diseases.

## Noncoding RNAs in cancer

Thousands of unique noncoding ribonucleic acids (ncRNAs) are present within cells, and they mediate functional molecules that regulate cellular processes, including chromatin remodeling, transcription, posttranscriptional modification, and signal transduction (12). ncRNAs include a variety of RNA types, such as short ncRNAs (sncRNAs) and long ncRNAs (lncRNAs). sncRNAs include ribosomal RNAs (rRNAs), which are involved in mRNA translation; transfer RNAs (tRNAs); spliced small nuclear RNAs (snRNAs); small-interfering RNAs (siRNAs); small nucleolar RNAs (snoRNAs), which are involved in rRNA modification; and RNAs involved in targeted translation and inhibition of gene silencing (microRNAs [miRNAs]) and Piwi-interacting RNAs (piRNAs) (13).

**Figure 1:**
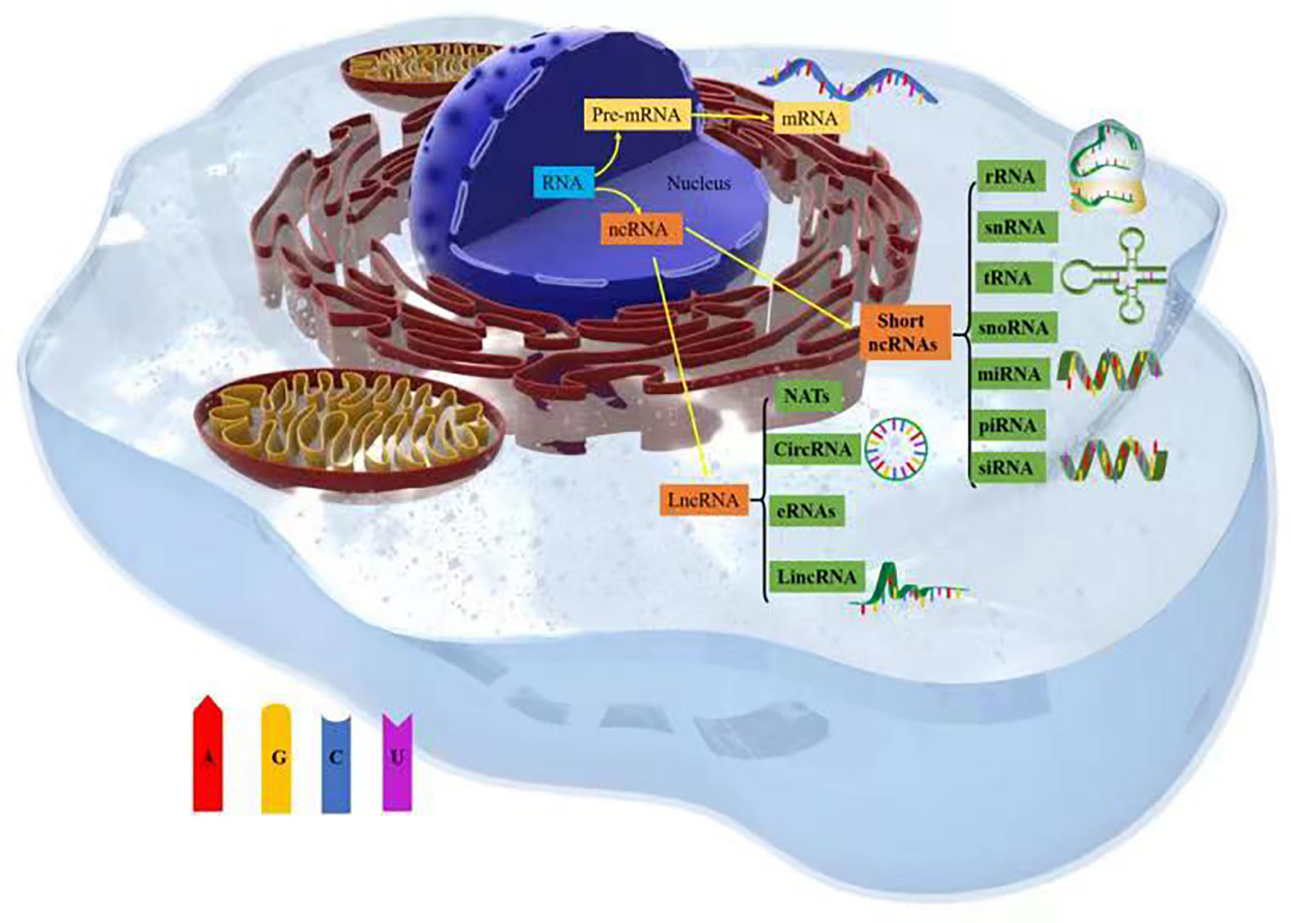
Types of noncoding RNAs (ncRNAs).

lncRNAs constitute a heterogeneous class of RNAs, including long intergenic ncRNAs (lincRNAs), circular RNAs (circRNAs), natural antisense transcripts (NATs), and enhancer RNAs (eRNAs) (14). These widespread ncRNAs are produced by the transcription of mammalian genomes and make up the majority of the transcribed genome, and only 1–2% of ncRNA transcripts are transcribed into proteins (15, 16). The roles played by miRNAs in cancer have been confirmed (17, 18), with thousands of papers reporting on the important roles that miRNAs play in cancer development and progression. For example, the degree to which miRNAs are conserved among species, expressed in different tissues and cell types, and participate in almost all biological processes (including cell cycle progression, growth, apoptosis, differentiation, and stress responses) and their ability to fine-tune gene expression by targeting multiple molecules have all been discussed (19). Notably, miR-130 and miR-494 can regulate cell survival and TNF-related apoptosis-induced ligand (TRAIL)–mediated therapy resistance in non-small-cell lung cancer (NSCLC) cell lines (20, 21). Therefore, ncRNAs play important roles in tumor cell metabolism (**Figure 1**).

## Lipid metabolism in tumors

**Figure 2:**
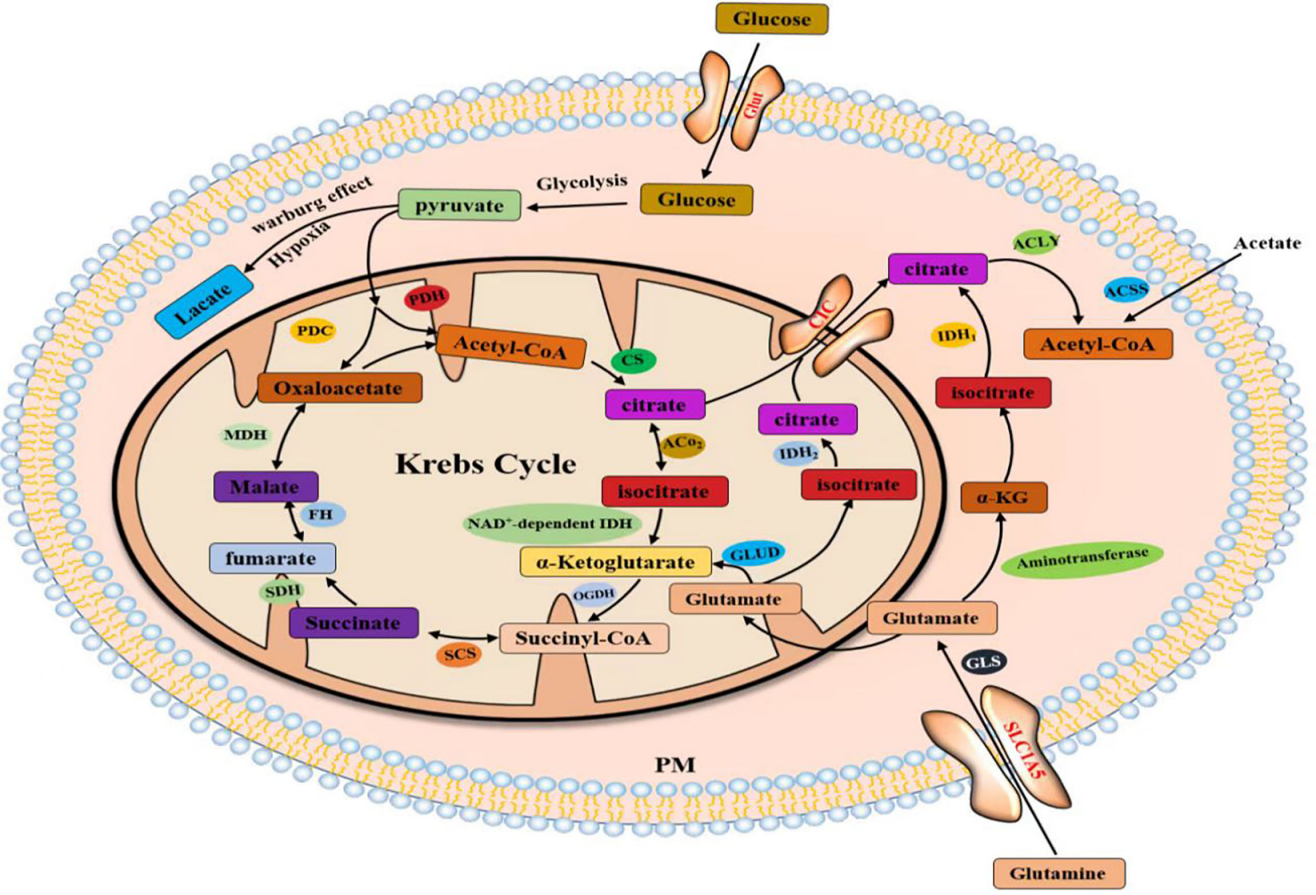
Acetyl-CoA production in the cytoplasm comes from three pathways.

**Figure 3:**
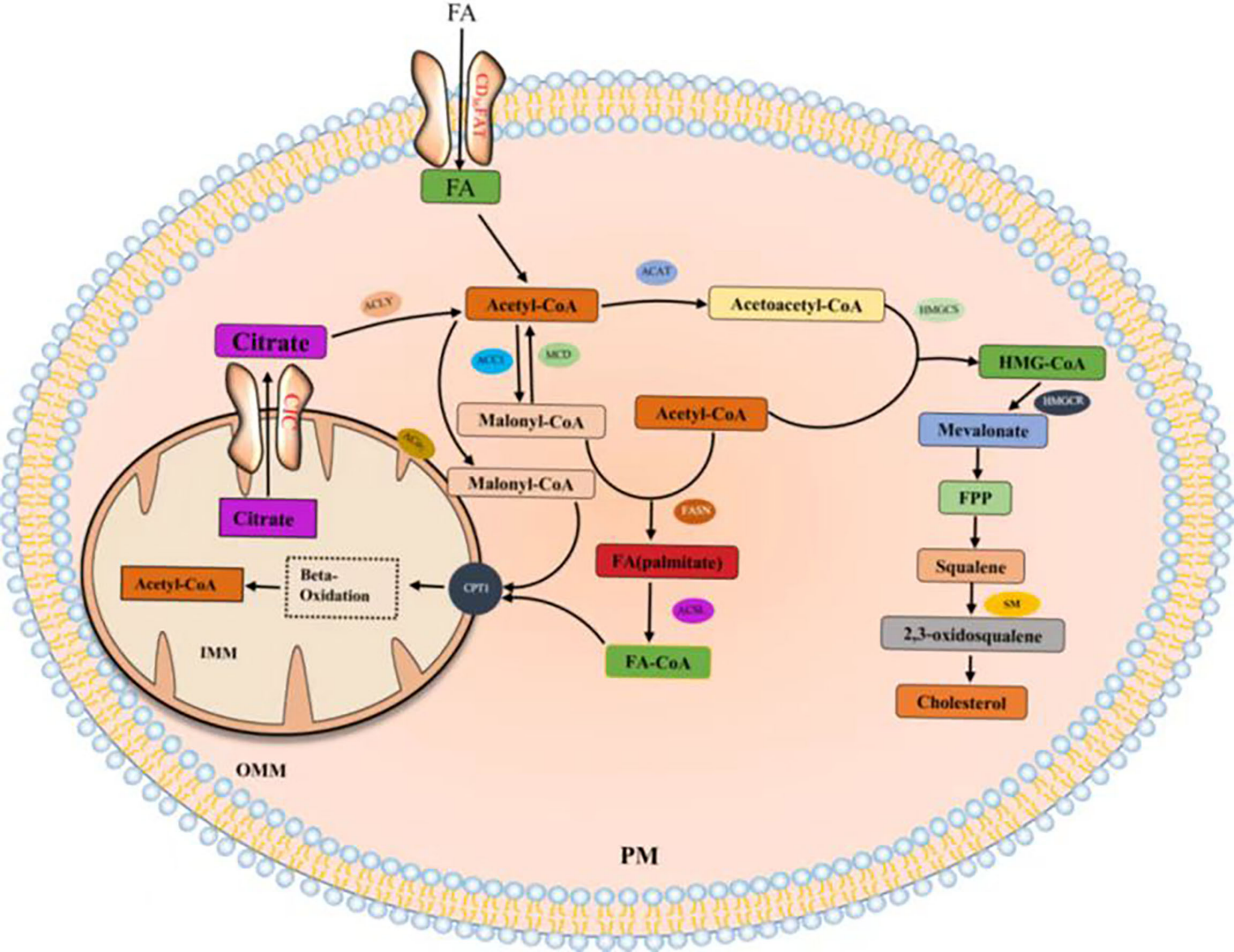
Fatty acids and cholesterol synthesis.

Citrate produced by glucose through glycolysis and Krebs cycle is mediated by a citrate carrier (CIC) into the cytoplasm and catalyzed by ATP-citrate lyase (ACLY) to generate acetyl-CoA (22, 23). Cytoplasmic acetyl-CoA can be extracted from acetic acid by acetyl-CoA synthase (ACSS) (24). Acetic acid (HAc) diffuses freely across cell membranes (25); in particular, under hypoxia, tumor cells capture acetate as an alternative carbon source (26–30). Citrate is obtained from glutamine, particularly from its carbon molecules, which contribute to citrate production (31, 32). Glutamine is transported into cells by a number of transporters, such as the extensively studied solute carrier family 1 neutral amino acid transporter member 5 (SLC1A5) (33). The catabolism of glutamine to glutamate is initiated by mitochondrial glutaminase (GLS). Glutamate is catalyzed by aminotransferase to produce α-ketoglutaric acid (α-kg) (34) and generate acetyl-CoA through a multistep enzymatic reaction or enter the mitochondrial tricarboxylic acid cycle to generate citrate, which is transported out of mitochondria by a CIC and catalyzed to produce acetyl-CoA through ACLY (**Figure 2**). Cytoplasmic acetyl-CoA is a substrate in fatty acid synthase (FAS). The interconversion of acetyl-CoA and malonyl-CoA in the cytoplasm is catalyzed by acetyl-CoA carboxylase (ACC) and malonyl-CoA decarboxylase (MCD) (35). ACC catalyzes the rate-limiting step in the fatty acid (FA) pathway. To enter a bioactive cell, an FA must be activated by acyl-CoA (FA-CoA) ligase (ACSL) to produce aliphatic FA-CoA (36, 37). FA-oxidation (FAO) is inhibited by the entry of FA-CoA into mitochondria, which is regulated by carnitine palmityl transferase 1 (CPT1) (37). Cholesterol synthesis begins with the condensation of two acetyl-CoA molecules mediated by cholesteryl transferase (ACAT) to form acetyl-CoA. Acetyl-acetyl-CoA is further condensed with a third acetyl-CoA molecule through 3-hydroxy-3-methylglutaryl-CoA synthase (HMGCS) to form 3-hydroxy-3-methylglutaryl-CoA (HMG-CoA). HMG-CoA reductase (HMGCR) is a glycoprotein located in the ER and catalyzes the rate-limiting step in cholesterol synthesis (38, 39). HMG-CoA is oxidized to 2,3-oxidized squalene by squalene monooxygenase (SQLE) through complex reactions. SQLE is the first oxidative step in cholesterol synthesis (39–41) (42–44) (**Figure 3**).

**Figure 4:**
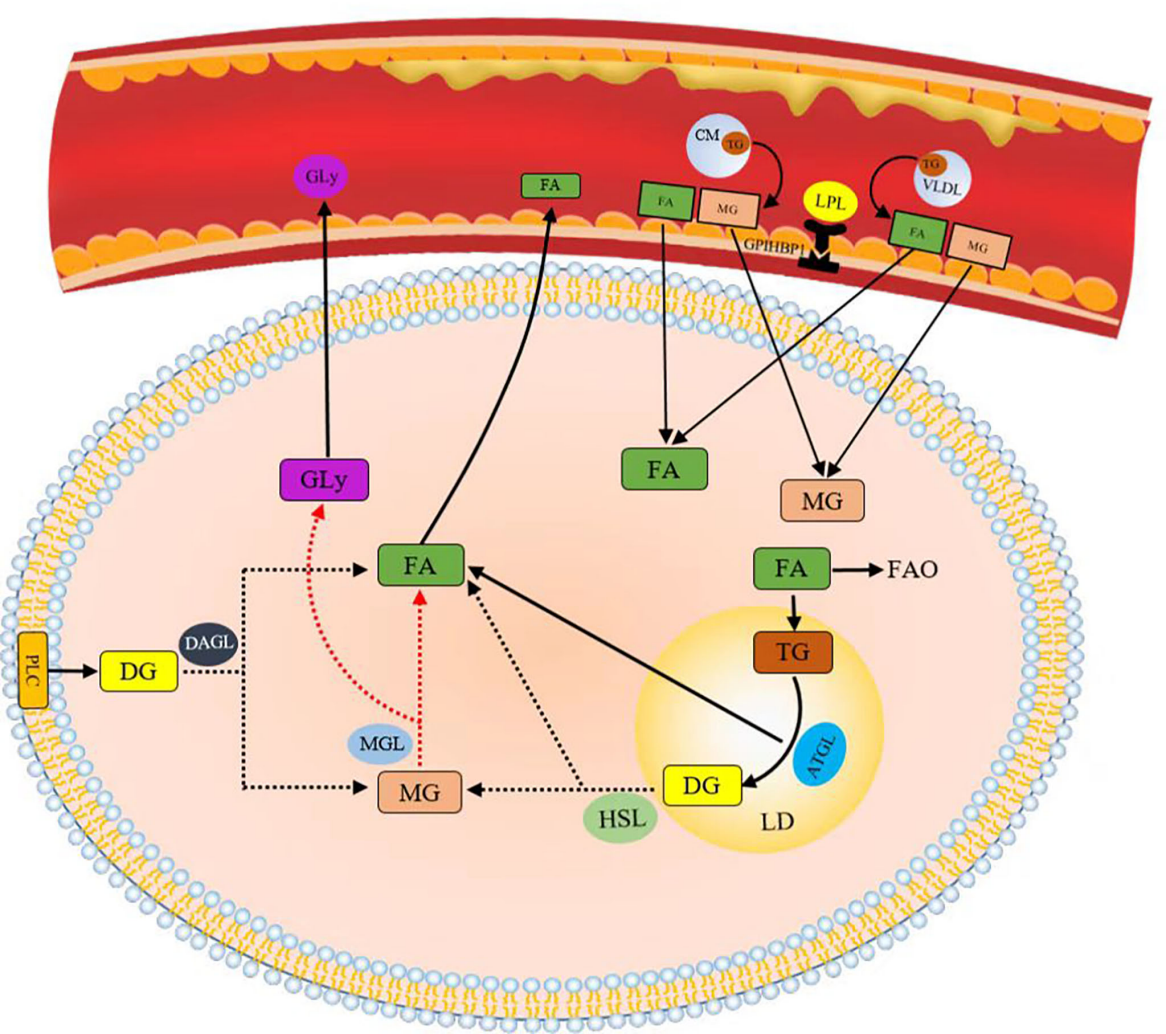
Lipids obtained by decomposition.

FA can be derived by intravascular and intracellular lipid decomposition. Lipoprotein lipase (LPL) is the rate-limiting component in plasma TG clearance and FA uptake by tissues (45). FA released by cyclic triTG hydrolysis can be absorbed by cells through CD36, a transmembrane channel protein with affinity for exogenous FA (46, 47). TG lipolysis is continuously performed by adipose triglyceride lipase (ATGL), hormone-sensitive lipase (HSL), and monoacylglycerol lipase (MGL) (48). ATGL plays an important catalytic role in the hydrolysis of TG to generate glycerol diesters (DGs) and FA in adipose and non-adipose tissues (49). HSL is the rate-limiting enzyme in the decomposition of DGs in adipose tissues (50). Phospholipase C (PLC) and diacylglycerol lipase (DGL) hydrolyze glycerol phospholipids to produce monoglycerol (MG) (**Figure 4**). Lipids constitute the basic structure of cell membranes. Phospholipids, glycolipids, and cholesterol are the main components of biofilms and significantly affect the fluidity of biofilms. At the cellular level, cholesterol in the cell membrane increases bilayer firmness and enhances its impermeability to water and ions. In addition to membrane formation, lipids are true second messengers that bind to specific sites to control channels and transporter gating. They also play a nonspecific role by changing the physical environment of channels and transporters, especially the protein–membrane interface (51). For example, phosphatidylinositol (4,5) diphosphate (PIP2) regulates K channels and changes ion channel permeability in sympathetic neurons (42). Lipid rafts are produced in the glycolipid-rich apical membrane of epithelial cells (43). They play an important role in post-Golgi transport, endocytosis, signal transduction, and many other membrane functions (44).

## Noncoding RNAs play different biological roles

Noncoding RNAs in the cytoplasm can regulate mRNA stability and translation efficiency and interfere with posttranslational modification of proteins. LncRNAs and circRNAs mainly perform two functions. First, they interact with RNA, including regulating mRNA stability as an endogenous rival RNA (ceRNA), inducing miRNA degradation to regulate mRNA activity, forming double-stranded RNA with mRNA to regulate mRNA stability and inhibit target mRNA translation. Second, they interact with proteins, including acting as molecular decoys for RNA-binding proteins (RBPs) to participate in mRNA degradation, regulating posttranslational modifications by covering up posttranslational modification (PTM) sites or PTM enzyme binding sites, and acting as translation proteins and coding peptides.

For target transcripts, the biological function of miRNAs includes two aspects. One is that miRNA induces the cleavage and degradation of target mRNA through complete base pairing with the 3′ untranslated region (3′ UTR) of mRNA; the other is that miRNA cannot perfectly complement the target mRNA, and its effect is only to restrain the translation of mRNA. The consistent end result of these two aspects is that the protein expression products of target mRNA are reduced.

In this section, we review the role of noncoding RNAs in tumor lipid metabolism through different biological functions and promote or inhibit malignant tumor expression. We can clearly see the role of noncoding RNAs in different tumors with different pathways (**Table 1**).

**Table 1:** Noncoding RNAs affect tumor lipid metabolism through different pathways.

biological function	Cancer types	Noncoding RNAs	Molecular axis	Result
**ceRNAs mode**	Salivary adenoid cystic carcinoma	LncRNA CASC9	LncRNA CASC9/miR- 146b-5p/ACLY	Promote
	Breast cancer	CircARL8B	CircARL8B/miR-653-5P/HMGA2	Inhibit
	Breast cancer	CircWHSC1	CircWHSC1/miR-195-5p/FASN/AMPK/ mTOR	Promote
	Esophageal squamous cell carcinoma	LINC00514	LINC00514/miR-378a-5p/ACCα,SPHK1	Promote
	Nasopharyngeal cancer	LINC02570	LINC02570/miR-4649-3p/FASN, SREBP-1	Promote
	Endometrial carcinoma	Linc-SNHG25	Linc-SNHG25/miR-497-5p/FASN	Promote
	Clear cell renal cell carcinoma	Circ-RPL23A	Circ-RPL23A/ miR-1233/ACAT2	Inhibit
	Primary cervical cancer	lncRNA LNMICC	lncRNA LNMICC/miR-190/ACC1,FASN	Promote
	Hepatocellular carcinoma	LncRNA HULC	LncRNA HULC/miR-9/PPARA/ACSL1/cholesterol/RXRA/ lncRNA HULC	Promote
**miRNA target mRNA**	Breast cancer	miR-22	miR-22/ACLY	Inhibit
	Breast cancer	miR-195	miR-195/ HMGCR	Inhibit
	Pancreatic cancer	miR-195	miR-195 /FASN/ Wnt	Inhibit
	Non-small-cell lung cancer	miR-320	miR-320/FASN	Inhibit
	Malignant meningiomas	miR-195	miR-195/FASN	Inhibit
	Hepatocellular carcinoma	miR-4310	miR-4310/ FASN, SCD1	Inhibit
	Hepatocellular carcinoma	miR-205	miR-205/ACSL1	Inhibit
	Hepatocellular carcinoma	miR-205	miR-205/ACSL4	Inhibit
	Esophageal squamous cell carcinoma	miR-133b	miR-133b/SQLE	Inhibit
	Leukemia cells	miR-23	miR-23/GLS	Inhibit
**molecular decoys for RBPs**	Non-small-cell lung cancer	LncRNA-CTD-2245E15.3	LncRNA-CTD-2245E15.3/ACC1/PC	Promote

### As endogenous competing RNAs to regulate downstream mRNA stability

LncRNAs and circRNAs act as molecular sponges of miRNAs and adsorb miRNAs through base complementation pairing, resulting in the antagonism between mRNAs and lncRNAs or circRNAs. We have found that a considerable number of noncoding RNAs are involved in the progression of malignancy through this mode. Unexpectedly, the endogenous competitive RNA regulation mode was combined with a positive feedback loop to regulate the malignant progression of tumors.

In salivary adenoid cystic carcinoma (SACC) cells, studies have shown that lncRNA tumor susceptibility gene 9 (CASC9) promotes the malignant phenotype acquired by SACC cells by regulating miR-146b-5p/ACLY axis activation (52). The lncRNA CASC9 binds to miRNA-146b-5p and negatively regulates its expression, and miR-146b-5p directly targets the 3’ untranslated region (UTR) of ATP-citric acid lyase (ACLY) to degrade ACLY. Moreover, lncRNA CASC9 upregulates ACLY expression through competitive binding with miR-146b-5p.

In breast cancer (BC) studies, circARL8B inhibited tumor lipid metabolism and cell proliferation through the miR-653-5P/HMGA2 axis (53). Moreover, circWHSC1 acted as an oncogene to expedite BC evolution by modulating the miR-195-5p/FASN/AMPK/mTOR pathway (54). CircWHSC1 acts as a competitive endogenous RNA by sponging miR-195-5p, and miR-195-5p directly targets fatty acid synthase (FASN). miR-195-5p overexpression inhibits FASN expression and activates its downstream AMPK pathway.

In esophageal squamous cell carcinoma (ESCC), LINC00514 promotes ESCC cell proliferation and invasion through the absorption of miR-378a-5p by sponges as competitive endogenous RNA, leading to the upregulation of adipoformation-related proteins, including acetyl-coenzyme (Co)A carboxylase α, SPHK1, FAS, and stearoyl-CoA desaturase 1 (55).

In addition, LINC02570 was upregulated in patients with clinically advanced nasopharyngeal cancer (NPC) and promoted NPC progression by upregulating FASN and sterol regulatory element-binding protein-1 (SREBP-1) through the adsorption of miR-4649-3p (56). Targeting SREBP-1 Mediated Lipogenesis as Potential Strategies for Cancer (57).

Recently, studies have shown that linc-SNHG25 positively regulates FASN expression and promotes endometrial carcinoma (EC) malignant development by sponging miR-497-5p (58).

According to recent research, the overexpression of circ-RPL23A has been shown to inhibit cell cycle progression, proliferation, migration, and invasion. miR-1233 directly targets cholesteryl transferase 2 (ACAT2) and is a target of circ-RPL23A, which inhibits clear cell renal cell carcinoma (ccRCC) progression by competitively binding miR-1233 and thus upregulating ACAT2 expression (48).

In primary cervical cancer lymph node metastasis, LNMICC significant downregulation of acetyl-CoA carboxylase 1 (ACC1), FASN in the deregulation of FA metabolism, and miR-190 exert inhibitory effects on LNMICC expression by directly binding to LNMICC (59).

In hepatocellular carcinoma (HCC), lncRNA HULC forms a positive feedback loop to promote adipogenesis and enhance tumor proliferation through the HULC/miR-9/PPARA/ACSL1/cholesterol/RXRA/HULC pathway (60).

**Figure 5:**
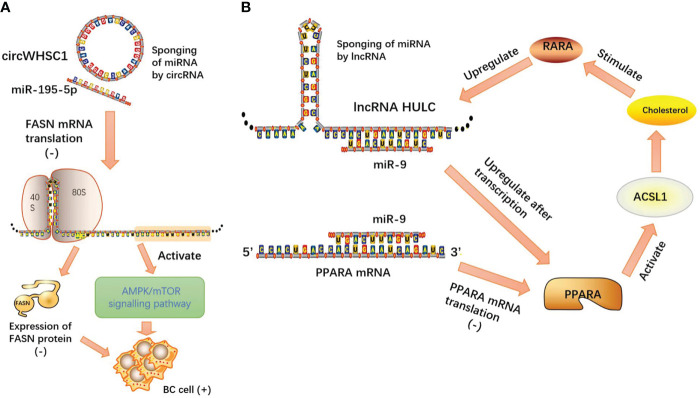
**(A)** CircWHSC1 acts as a competitive endogenous RNA by sponging miR-195-5p, and miR-195-5p directly targets FASN mRNA. miR-195-5p overexpression inhibits FASN expression and activates its downstream AMPK pathway; **(B)** lncRNA HULC forms a positive feedback loop to promote adipogenesis and enhance tumor proliferation through the HULC/miR-9/PPARA/ACSL1/cholesterol/RXRA/HULC pathway.

LncRNAs and circRNAs, as two kinds of ncRNAs, exert multitudinous biological functions and act as molecular sponges, relying on microRNA response elements (MREs) to competitively target microRNAs (miRNAs), thereby attenuating the degradation or inhibition of miRNAs to their own downstream protein-coding target genes and regulating the initiation and progression of neoplasms. Competing endogenous RNAs (ceRNAs) play an important role in the progression of tumor malignancy. Based on the theory of this model, we can design lncRNA-, circRNA-, or microRNA-targeted gene drugs to control the tumor process, and at the same time, the existence of tumors can be diagnosed by circulating tumor cell detection (**Figure 5**).

### miRNAs directly target mRNA of lipid-metabolizing enzymes

The classical biological function of miRNA is to inhibit the translation of mRNA and to reduce the protein expression products of target mRNA by pairing with the base of mRNA. In addition, we unexpectedly found that miRNAs can target mRNAs of lipid metabolism enzymes through another miRNA.

In breast cancer research, miR-22 inhibits FA synthesis and elongation in tumor cells by targeting ACLY and FA elongase 6 (61). Moreover, HMGCR and FASN are direct targets of hsa-miR-195 in BC cells. hsa-miR-195 has been shown to target genes involved in *de novo* adipogenesis and to inhibit cell proliferation, migration, and invasion (62).

In pancreatic cancer-related studies, microRNA-195 directly targets the FAS enzyme and negatively regulates the expression of FAS, miRNA-195 overexpression inhibits the proliferation and invasion of pancreatic cancer cells, and miRNA-195 inhibits Wnt signaling in pancreatic cancer cells. The results showed that miRNA-195 inhibits pancreatic cancer cell proliferation and invasion by regulating the FASN/Wnt signaling pathway (63).

In lung cancer studies, it was found that miR-320 directly targets FASN to inhibit the growth, migration, and invasion of NSCLC cells and is significantly correlated with TNM classification and metastasis (64).

We found that FASN was significantly upregulated in malignant meningiomas. miR-195 directly targeted FASN. miR-195 has been demonstrated to play a tumor suppressive role in the occurrence and progression of malignant meningioma by targeting FASN (65).

By inhibiting FASN and stearyl CoA desaturase 1 (SCD1)–mediated lipid synthesis, miR-4310 inhibited HCC cell proliferation, migration, and invasion *in vitro* and inhibited HCC tumor growth and metastasis *in vivo*. These results indicate that miR-4310 plays an important role in HCC tumor growth and metastasis by targeting FASN and SCD1-mediated lipid synthesis pathways (66).

Studies have shown that miR-205 mediates the dysregulation of HCC lipid metabolism by targeting acyl-CoA long-chain family member 1 (ACSL1) mRNA, and ACSL1 is significantly affected by ncRNA-mediated lipid metabolism in HCC cells (67). In addition, miR-205 inhibited acyl-CoA long-chain family member 4 (ACSL4) expression at the mRNA and protein levels by targeting its 3’UTR. miR-205 expression promotes abnormal lipid metabolism in HCC by targeting ACSL4 (68).

In human ESCC cells and tissues, miR-133b expression was reported to be downregulated. SQLE is a direct downstream target gene of miR-133b. Exogenous miR-133b expression and SQLE knockdown reduced the rate of the epithelial–mesenchymal transition (EMT) in ESCC cells *in vitro*. These outcomes indicate that miR-133b–dependent SQLE potentially plays a key role in ESCC metastasis (69).

miR-23 targets GLS mRNA and inhibits GLS protein expression. The overexpression of miR-23a has been reported to impair glutamine metabolism and induce apoptosis in leukemia cells (70).

**Figure 6:**
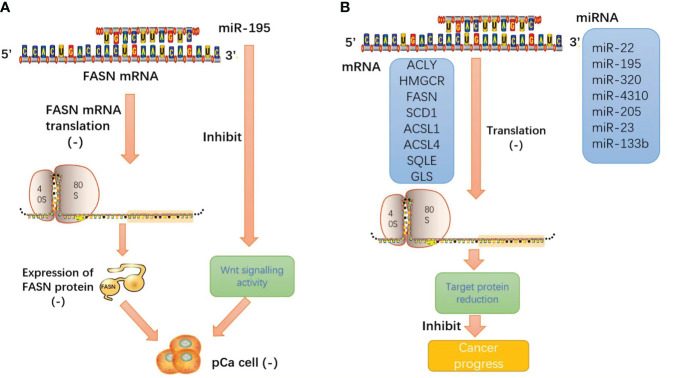
**(A)** miRNA-195 inhibits pancreatic cancer cell proliferation and invasion by regulating the FASN/Wnt signaling pathway; **(B)** miRNA induces the cleavage and degradation of target mRNA through base pairing with the 3′ untranslated region (3′ UTR) of mRNA. The result is that the protein expression products of target mRNA and the malignant behavior of tumors are inhibited.

We consistently found that these miRNAs inhibit the enzyme function of lipid metabolism and reduce the production of metabolic enzymes through base complementary pairing with the mRNA of the enzyme. miRNA plays an important role in tumor lipid metabolism and inhibits malignant progression through its classical biological functions. Such intuitive effects make people have great interest in the research and development of miRNA mimics, especially the products harmless to human body after biological processing, which will provide a bright future for tumor treatment (**Figure 6**).

### Noncoding RNAs act as molecular decoys for RNA-binding proteins

Some specific RNA-binding domains of noncoding RNAs can be recognized and interact with RNA-binding proteins and are involved in various posttranscriptional regulatory processes, such as RNA shearing, transport, sequence editing, intracellular localization, and translation control, which play a role in the malignant biological behavior of tumors to a certain extent.

In recent studies on NSCLC, LncRNA-CTD-2245E15.3 exerts its carcinogenic function by binding ACC1 and pyruvate carboxylase (PC), which are key anabolic factors in biomolecule synthesis in rapidly proliferating tumor cells (71).

**Figure 7:**
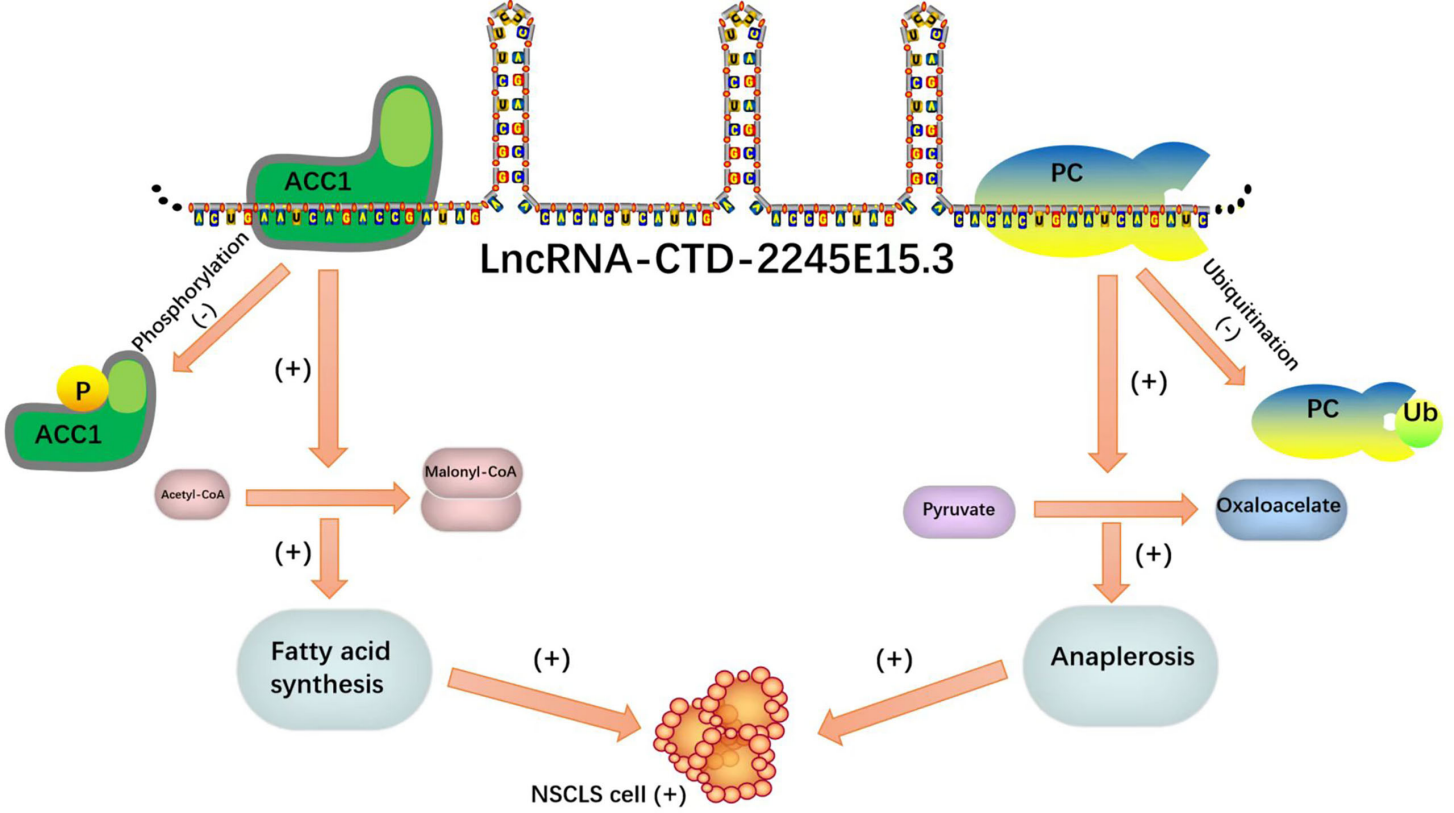
As molecular decoys for RNA binding proteins (RBPs).

Lipid metabolism enzymes can bind to RNA binding proteins as ncRNA molecular decoys, inhibiting the ability of lipid metabolism enzymes to promote the progression of tumor malignancy. Such a binding mode can be a reasonable direction for tumor gene therapy. It is a good idea to design special probes to enhance the binding of ncRNA and RNA binding protein (RBP) for tumor targeted therapy (**Figure 7**).

### Noncoding RNAs regulate the expression of lipid metabolism enzymes

**Figure 8:**
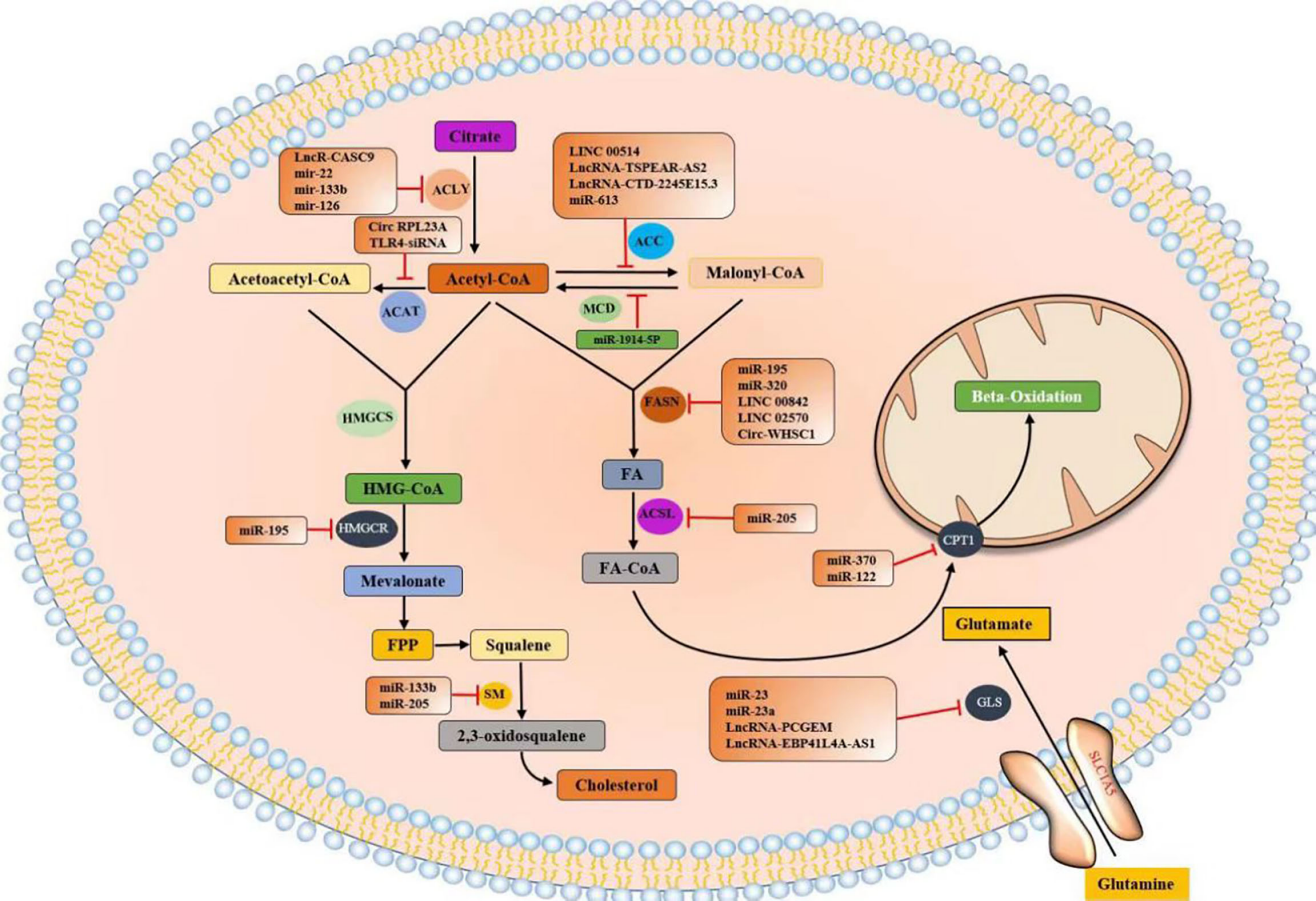
Noncoding RNAs regulate the expression of lipid metabolism enzymes.

Tumor metabolic reprogramming mainly depends on the changes of metabolic enzymes, and noncoding RNA plays an important role in the regulation of tumor lipid metabolic enzymes. In order to have a more intuitive understanding of the regulation of various lipid metabolism enzymes, we used enzymes as clues to summarize the interference of various noncoding RNAs in tumor malignant biological behavior by regulating the expression level of tumor lipid metabolism enzymes and presented them in the form of tables and figures (**Tables 2**, **3** and **Figure 8**). We can clearly understand the leading role of noncoding RNA in tumor lipid metabolism, which provides a theoretical basis for noncoding RNA as a key molecule in targeted therapy and a solid theoretical basis for precision medicine in the future.

**Table 2:** Direct signaling pathways of Noncoding RNAs in tumor lipid metabolism.

Lipid metabolism enzymes	Cancer types	Noncoding RNAs	Molecular axis	Result
ACLY	Breast cancer	miR-22	miR-22/ACLY	Inhibit
	Malignant mesothelioma	miR-126	miR-126/ISR1/ACLY	Inhibit
	Gastric cancer	miR-133b	miR-133b/ACLY	Inhibit
	Salivary adenoid cystic carcinoma	LncRNA CASC9	LncRNA CASC9/miR- 146b-5p/ACLY	Promote
ACC	HepG2 cells	miR-613	miR-613/ACC/SREBP-1c /FASN/ChREBP	Inhibit
	Primary cervical cancer	lncRNA LNMICC	lncRNA LNMICC/ACC1	Promote
	Breast cancer	CircARL8B	CircARL8B/miR-653-5P/HMGA2	Inhibit
	Non-small-cell lung cancer	LncRNA-CTD-2245E15.3	LncRNA-CTD-2245E15.3/ACC1	Promote
	Esophageal squamous cell carcinoma	LINC00514	LINC00514/ACCα,SPHK1	Promote
	Colorectal cancer	lncRNA TSPEAR-AS2	lncRNA TSPEAR-AS2/ ACC1, FASN	Promote
FASN	Pancreatic cancer	miR-195	miR-195 /FASN/ Wnt	Inhibit
	Pancreatic ductal adenocarcinoma	LINC00842	LINC00842/FASN	Promote
	Pancreatic cancer	miR-33a	miR-33a/AMPK/ mTOR/ FASN	Inhibit
	Nasopharyngeal cancer	lncRNA HOTAIR	lncRNA HOTAIR/ FASN	Promote
	Nasopharyngeal cancer	LINC02570	LINC02570/ miR-4649-3p/ FASN, SREBP-1	Promote
	Non-small-cell lung cancer	miR-320	miR-320/FASN	Inhibit
	Malignant meningiomas	miR-195	miR-195/FASN	Inhibit
	Endometrial carcinoma	Linc-SNHG25	Linc-SNHG25/miR-497-5p/FASN	Promote
	Hepatocellular carcinoma	miR-4310	miR-4310/ FASN, SCD1	Inhibit
	Colorectal cancer	LncRNA DNAJC3-AS1	LncRNA DNAJC3-AS1/ EGFR/PI3K/AKT/NF-κB/FASN	Promote
	Breast cancer	CircWHSC1	CircWHSC1/miR-195-5p/FASN/AMPK/ mTOR	Promote
ACSL	Hepatocellular carcinoma	miR-205	miR-205/ACSL1	Inhibit
	Hepatocellular carcinoma	LncRNA HULC	LncRNA HULC/miR-9/PPARA/ACSL1/cholesterol/RXRA/ lncRNA HULC	Inhibit
	Hepatocellular carcinoma	miR-205	miR-205/ACSL4	Promote
ACAT	Clear cell renal cell carcinoma	Circ-RPL23A	Circ-RPL23A/ miR-1233/ACAT2	Inhibit
HMGCR	Breast cancer	miR-195	miR-195/ HMGCR,FASN, CYP27B1	Inhibit
SQLE	Esophageal squamous cell carcinoma	miR-133b	miR-133b/SQLE	Inhibit
	Prostate cancer	miR-205	miR-205/SQLE	Inhibit

**Table 3:** Indirect signaling pathways of Noncoding RNAs in tumor lipid metabolism.

Lipid metabolism enzymes	Cancer types	Noncoding RNAs	Molecular axis	Result
GLS	Prostate cancer	miR-23	c-Myc/miR-23/GLS	Promote
	Leukemia cells	miR-23	miR-23/GLS	Inhibit
	Prostate cancer	LncRNA PCGEM	LncRNA PCGEM/GLS, ALCY,FASN	Promote
	Colorectal cancer	LncRNA EPB41L4A-AS1	LncRNA EPB41L4A-AS1/GLS1	Promote
CPT1	HepG2 cells	miR-370	miR-370/ miR-122/ CPT1α	Inhibit

At present, the most studied lipid metabolism enzymes are ACLY, ACC, and FASN. ACLY is a cytoplasmic enzyme that catalyzes citric acid to produce acetyl-CoA and is an important component in the endogenous biosynthesis of FA and cholesterol (72). In the study of various tumors, including breast cancer, malignant mesothelioma, gastric cancer, salivary adenoid carcinoma, and noncoding RNA can affect the expression of lipid metabolism enzymes by directly binding to ACLY or by classical ceRNA mode, thus changing the malignant process of tumors. Relevant noncoding RNA mimics or inhibitors can be studied for molecular therapy of tumors.

ACC is a critical rate-limiting enzyme for FASN (73). In colorectal cancer (CRC) studies, *in vitro* experiments have shown that lncRNA TSPEAR-AS2 knockdown can reduce the TG content and ACC1 and FASN expressions in CRC cells. These outcomes suggest that lncRNA TSPEAR-AS2 can regulate FA metabolism in CRC (74). In the studies of primary cervical cancer, breast cancer, non-small-cell lung cancer (NSCLC), and esophageal carcinoma (ESCC), noncoding RNA can regulate ACC expression and affect tumor proliferation in various ways.

FASN is a key enzyme for the *de novo* synthesis of FA, plays an important role in lipid metabolism, and is associated with tumor-related signaling pathways (75). FASN plays a central role in lipid metabolism, so it has been extensively studied in pancreatic cancer, pancreatic ductal adenocarcinoma, NPC, and seven other tumors. Most of the research results show that noncoding RNA plays a role by binding miRNA or RBP or directly binding mRNA of lipid metabolism enzymes, and interferes with the biological behavior of tumor malignancy.

## Future perspectives

### Current research achievements and deficiencies

At present, we clearly understand the reactions involved in tumor lipid metabolism and the influence of ncRNAs on lipid metabolism-related enzymes, which change tumorigenesis and tumor development processes. These results provide us with a profound understanding of the influence of ncRNAs on tumor lipid metabolism and lay a solid foundation for the study of tumor lipid metabolism. Is this the end of research? Of course not. There are still unanswered questions.

First, ncRNAs are involved in the regulation of lipid metabolism in the cytoplasm, but their source is the nucleus. How do various ncRNAs cross the nuclear membrane into the cytoplasm? Current research has focused on the length of ncRNAs and related protein mediations (76, 77). Whether there are other factors, such as the sequence of ncRNA bases and the proportion of various bases, needs further investigation.

Second, everything in life has a life cycle; thus, are ncRNAs immortal? How are they regulated and degraded? The four currently known degradation pathways, namely microRNA, m^6^A, ribonuclease L (RNase L), and special structure-mediated degradation (78–81), remain to be further studied.

However, there are few studies on the regulation of several lipid metabolism enzymes by ncRNAs in the tumor metabolism process, such as MCD (82), ACSL (60, 67, 68), ACAT (48, 83), HMGCS, and HMGCR (62). These lipid metabolism enzymes will become research objects in the future.

Finally, some current studies have demonstrated that ncRNAs can directly act on the activities of lipid metabolism enzymes or their encoding gene regulatory enzymes. Whether there are other unknown signaling pathways that serve as a bridge between ncRNAs and lipid metabolism enzymes remains to be further investigated.

### Future research directions for noncoding RNAs

Because ncRNAs can affect tumor processes by regulating lipid metabolism enzymes, in the future, we can study the unique role of ncRNAs from the four aspects of disease prevention, detection, diagnosis, and treatment.

In the future, as in the prevention of many diseases, ncRNA vaccines will be the first line of defense for various incurable diseases, including tumors. At present, the world is facing the challenge of a novel coronavirus. Scientists have developed relevant vaccines, such as the BNT162b2 mRNA COVID-19 vaccine (84) and the mRNA-1273 vaccine (85, 86), which have achieved good immune effects in clinical applications. A good tumor response rate was shown in clinical studies using mRNA vaccines in patients with advanced melanoma (87, 88). We believe that the advent of ncRNA vaccines will play an important role in tumor preventive immunity.

In terms of inspection and diagnosis, we will focus on the examination of patient body fluids, such as whole blood, plasma, serum, gastroenteric fluid, and urine, to detect and quantify the substances and related ncRNAs released by tumor cells to accurately diagnose diseases combined with other relevant information. Currently, circRNA and RNA splicing variants have been reported in lung cancer as cancer biomarkers and have achieved satisfactory results in evaluating therapeutic responses (89). Moreover, hsa_circ_002059 and hsa_circ_0000419 have been confirmed as potential novel and stable biomarkers for the diagnosis of gastric cancer (90, 91), and the role of hsa_circ_0004585 in CRC progression has been confirmed, indicating its potential as a biomarker for diagnosis (92). A recent survey showed that a total of 33 circulating miRNAs and six different panels of circulating miRNAs have been described for their diagnostic performance in EC diagnosis. A total of seven circulating miRNAs and one panel of circulating miRNAs have been associated with clinical and prognostic factors in EC (93). In a variety of tumor studies, significant progress has been made in the use of ncRNAs in extracellular fluid as cancer biomarkers (94).

Current tumor treatments include surgical excision, chemotherapy, and monoclonal antibody therapy. Based on the understanding of ncRNA, it has great potential as a gene-targeted therapy. At present, in lung cancer research, the effectiveness of a combination of mRNA vaccines and radiotherapy to eradicate solid tumors has been scientifically confirmed (95). An mRNA vaccine has also been reported to be safe and effective in inducing antigen-specific T-cell immunity in clinical studies of gastrointestinal tumors (96). For tumor resistance to chemotherapeutic drugs, there is a considerable amount of data to prove that noncoding RNA can enhance tumor sensitivity to chemotherapy. To date, a number of advanced miRNA detection methods with high specificity and sensitivity have been developed, such as chromatography-based methods and mass spectrometry-based nanomaterial-based methods. miRNA detection methods are going to focus on facilitating the development of noninvasive diagnosis and inhibiting cancer drug resistance (97). In a recent study, the combined use of microRNAs and long noncoding RNAs with chemotherapeutic compounds, as well as the induction of suicide genes, provided an innovative therapeutic approach for the management of glioblastoma (GBM) (98). It has been reported that miR-532–3p can directly target E26 oncogene homolog 1 (ETS1) and transglutaminase 2 (TGM2) to promote early apoptosis through the ETS1/TGM2 axis-mediated Wnt/β-catenin signaling pathway, and it activates the p53 signaling pathway and enhances the sensitivity to cisplatin and 5-FU in CRC treatment (99). Similarly, it was discovered that miR-365–3p inhibited the downstream expression of keratin 16 (KRT16) to enhance 5-FU–induced cytotoxicity through the c-Met/Src signaling pathway and curbed oral squamous cell carcinoma (OSCC) metastasis and cancer stemness (100). Therefore, ncRNAs not only can serve as targeted therapeutic sites but also can enhance the therapeutic effect of chemotherapy-resistant tumors and can also be combined with radiotherapy for the radical treatment of tumors. In the future, the development of ncRNA therapy will become a hot direction and shoulder a major task in the medical field.

## Conclusions

In this paper, we systematically introduce the metabolism and function of lipids and their changes in tumorigenesis and explain the relationship between ncRNAs and tumor lipid metabolism, including how ncRNAs regulate tumor lipid metabolism and regulatory pathways. Finally, the progress of ncRNAs in disease prevention, detection, diagnosis, and treatment is discussed. Our goal is to take advantage of the characteristics and advantages of ncRNAs and use them in multiple stages of the disease. Current scientific and technological methods include gene knockout, molecular targeting, isothermal amplification-based methods, nanomaterial-based methods, chromatography-based methods, and mass spectrometry-based methods. We are very confident that ncRNAs can be incorporated into the diagnosis and treatment process to target rivals in the stage of disease detection and diagnosis, weaken rivals in the stage of prevention, and defeat rivals in the stage of treatment, providing a new platform for all-in-one tumor therapy.

## Author contributions

YW, QL, SW and B-JW collected the related reports and drafted the manuscript. LX, X-XH and Y-BX revised the manuscript. YJ, HH, Q-SF, J-WW, QW, LQ, and T-TC participated in designing the review. All authors contributed to the article and approved the submitted version.

## Funding

This work was supported by the Natural Science Research Project of Higher Education in Anhui Province (KJ2021A0857); the National Natural Science Foundation of China (81902515); Excellent Youth Talent Support Program (Key) of Anhui Province (gxyqzd2020029); Introducing Talents Natural Science Foundation of The First Affiliated Yijishan Hospital of Wannan Medical College (YR202006); the Natural Science Research Project of Higher Education in Anhui Province (KJ2019A0429); and the Natural Science Research Project of Higher Education in Anhui Province (KJ20190412).

## Acknowledgments

We would like to acknowledge the reviewers for their helpful comments on this paper.

## Conflict of interest

The authors declare that the research was conducted in the absence of any commercial or financial relationships that could be construed as a potential conflict of interest.

## Publisher’s note

All claims expressed in this article are solely those of the authors and do not necessarily represent those of their affiliated organizations, or those of the publisher, the editors and the reviewers. Any product that may be evaluated in this article, or claim that may be made by its manufacturer, is not guaranteed or endorsed by the publisher.
